# Sustained breastfeeding associations with brain structure and cognition from late childhood to early adolescence

**DOI:** 10.1038/s41390-025-04086-x

**Published:** 2025-05-17

**Authors:** Jonatan Ottino González, Miguel Angel Rivas Fernández, Sevan Esaian, Vidya Rajagopalan, Mustapha Bouhrara, Michael I. Goran, Shana Adise

**Affiliations:** 1 Division of Endocrinology, Diabetes and Metabolism, Department of Pediatrics, Children’s Hospital Los Angeles, Los Angeles, CA USA; 2 Division of Cardiology, Department of Pediatrics, Children’s Hospital Los Angeles, Keck School of Medicine, University of Southern California, Los Angeles, CA USA; 3 Laboratory of Clinical Investigation, National Institute on Aging, NIH, Baltimore, MD USA

## Abstract

**Background:**

While breastfeeding benefits early child neurocognition, its influences into adolescence, a period of intense brain remodeling and heightened mental health risk, remain unclear.

**Methods:**

Breastfeeding and neurocognitive longitudinal associations were explored over a two-year period in the Adolescent Brain Cognitive Development (ABCD) Study® (*n*_baseline_ = 5098, ages 9–10, 49% female; *n*_follow-up_ = 3810, ages 11–12, 48% female). Breastfeeding duration was reported as never breastfed (15.8%), 1–6 months (34.6%), 7–12 months (26.4%), and >12 months (23.1%). MRI-derived estimates of cortical thickness, surface area, and cortical myelin were calculated across 148 brain regions alongside fluid cognition measures. Linear mixed-effects models tested the influence of breastfeeding duration and its interaction with age on neurocognitive outcomes. Significant cortical thickness and surface area associations were explored for cortical myelin differences. Parallel mediation analyses examined whether cortical features mediated the breastfeeding-fluid cognition relationship.

**Results:**

Breastfeeding duration was positively associated with cortical thickness (31 regions), surface area (45 regions), and fluid cognition (all *p* values < 0.05), and with greater cortical myelin in four regions and increases by follow-up in 12 regions (all *p* values < 0.05). Surface area mediated the breastfeeding-fluid cognition link (*β* = 0.008, CI_boot_95% = 0.005, 0.012).

**Conclusions:**

These findings emphasize the importance of extending breastfeeding practices for optimal adolescent neurocognition.

**Impact:**

- Does breastfeeding influence neurocognition during early adolescence, and does it impact neurocognitive development at this stage?
- In this longitudinal study, breastfeeding demonstrated dose-dependent, lasting positive influences on neurocognition that remained stable over a 2-year period spanning late childhood to early adolescence.
- Specifically, individuals who were breastfed longer showed increased cortical thickness, surface area, cortical myelin, and fluid cognition, predictors of positive outcomes in later life, including physical and mental health.
- Our findings highlight the importance of breastfeeding and support its extended practice for optimal neurodevelopment and potential late-life benefits.

## Introduction

Neurodevelopment encompasses substantial brain changes, with the first years of life being especially critical.^1^ Research indicates that, with the exception of very preterm infants who often require fortified breast milk,^2^ breast milk provides all the necessary nutrients to support optimal neurodevelopment.^3^ As such, exclusive breastfeeding is recommended for the first 6 months of life and advised up to 2 years due to additional neurocognitive benefits.^4^ However, breastfeeding rates have been alarmingly decreasing, possibly due to societal, cultural, and economic challenges.^5^ This is concerning as formula milk, which has been negatively linked to neurocognitive outcomes, is becoming the primary alternative.^6^ Given the critical role of early nutrition on neurodevelopment, there is a dire need to understand breastfeeding’s lasting influences, especially during adolescence, an underexplored developmental window.

Research has shown that exclusive and prolonged breastfeeding is positively linked to neurocognitive outcomes strongly predictive of academic and professional success, and physical and mental health.^7,8^ Concretely, breastfeeding has been associated with greater cortical thickness and surface area, enhanced white matter myelination, and improved executive function.^9–13^ One proposed mechanism lies in the composition of breast milk. Several of its components, such as lactoferrin, choline, fatty acids, and growth factors, are thought to be key in assisting critical postnatal neurodevelopmental processes for brain structure and function, such as synaptogenesis, gliogenesis, and myelination.^14–18^

Despite the well-established benefits of breastfeeding, conflicting findings exist.^10,11,19–22^ These inconsistencies may stem from variations in study design, sample characteristics, covariate selection, and the brain metrics used. For instance, some research has relied on whole-brain cortical thickness or surface area averages, which may obscure localized effects. Additionally, studies have been limited to early childhood and cross-sectional designs. Thus, leaving unanswered questions on differences in neurodevelopmental trajectories into adolescence, a period of intense brain remodeling and increased mental health risk.^23^ Moreover, although cortical myelin has been related to both breastfeeding^17^ and cortical thickness and surface area,^24,25^ its potential role as a mechanism underlying changes in these cortical features relative to breastfeeding remains unclear. Similarly, the interrelationship among breastfeeding duration, cortical features, and cognition requires further exploration. Given the current low rates of breastfeeding, the inconsistencies in the literature, and the numerous gaps, there is a need to investigate these associations, which may help to promote best early nutritional practices for optimal neurodevelopment.

The current study examined the longitudinal association between breastfeeding duration and regional cortical features, specifically cortical thickness, surface area, and cortical myelin, along with proxies of executive function, such as fluid cognition,^26^ over a 2-year period spanning late childhood and early adolescence (i.e., ages 9–12-years-old). Leveraging data from the Adolescent Brain Cognitive Development (ABCD) Study®, we aimed to determine whether breastfeeding duration has lasting effects on regional cortical thickness, surface area, and fluid cognition into adolescence and to what extent these associations change over time. We expect positive relationships between breastfeeding duration and neurocognitive outcomes (e.g., greater cortical thickness) independent of time. Also, we hypothesize that longer breastfeeding duration will correlate with markers that suggest optimal neurodevelopment, such as increases in surface area from baseline to follow-up. Additionally, we examined the extent to which regions showing cortical thickness and surface area differences also exhibit variations in cortical myelin. Lastly, we explored whether changes in these cortical features mediated the link between breastfeeding duration and fluid cognition.

## Methods

### Study design and participants

The ABCD Study® is a 10-year, 21-site study that started between 2016 and 2018 when youth were aged 9 and 10 years old. Recruitment was tailored to match the demographic population of the US Census. The ABCD Study® is responsible for obtaining caregiver consent and youth assent. The overall protocol was approved by the centralized Institutional Review Board at the University of California, San Diego. This manuscript used data from the 5.1 release (10.15154/z563-zd24) and focused on data collected at baseline and follow-up (2018–2020). While the ABCD Study® had minimal exclusions, the current analysis applied additional exclusions (e.g., breastfeeding reported by biological mothers only, no siblings, etc.). MRI assessments at follow-up were missing for some participants due to a pause in data collection during the COVID-19 lockdown. Although fluid cognition data was still collected remotely for some participants, we only considered in-person assessments to prevent the effects of remote assessments on cognitive performance. Details can be found in the Supplementary Material section (see Supplementary Methods).

### Demographics and physical characteristics

At baseline, caregivers reported on the youth’s race, sex at birth, highest household education, prenatal substance exposure, and perinatal issues. Information on the youth’s handedness, visual acuity, and pubertal stage was also obtained. Additional details are reported in the Supplementary Methods.

### Breastfeeding duration

Caregivers retrospectively reported breastfeeding duration at baseline and follow-up at year 3. Breastfeeding reports were cross-referenced to ensure accuracy (see Supplementary Methods). As breastfeeding duration was right-skewed, this variable was recoded into simpler numeric categories: 0 = never breastfed (only formula), 1 = 1–6 months, 2 = 7–12 months, 3 = more than 12 months. These cut-offs align with recent works in this cohort.^11,20^

### Image acquisition and preprocessing

The ABCD Study® collected T1-weighted (T1w) and T2-weighted (T2w) data using 31 different 3T scanners. Estimates of regional cortical thickness (in millimeters, mm) and surface area (mm^2^) were extracted with the Destrieux atlas (148 regions) using Freesurfer (version 7.1.1).^27^ Cortical myelin was estimated by dividing the intensity of gray matter vertices located 0.02 mm above the gray/white matter limit from T1w and T2w images.^28^ Poor-quality data flagged by the Data Analysis, Informatics, and Resource Center were removed (see Supplementary Methods). LongCombat (v0.0.0.9)^29^ was used to remove scanner influences from MRI-derived cortical features while preserving the effects of breastfeeding duration, confounders (age, education, prematurity, handedness, sex), and repeated measures.

### Fluid cognition

The National Institutes of Health Toolbox Cognitive Battery (NIH Toolbox) was administered at baseline and follow-up.^30^ Site-related effects (*n* = 21 sites) were removed from the uncorrected standardized scores obtained in the Flanker Inhibitory Control and Attention, Picture Sequence Memory, and Pattern Comparison Processing Speed tasks using longCombat.^29^ Scores were averaged into a fluid cognition composite (see Supplementary Methods).

### Statistical analyses

All statistical analyses were conducted using R (version 4.3.1). This manuscript used the *tidyverse* (version 2.0)*, data.table* (version 1.15.4), *summarytools* (version 1.0.1), and *effectsize* (version 0.8.9) for data wrangling, summarization, visualization, and standardization of reported coefficients. Outliers ( ± 3 SD) were removed from severely skewed, heavily-tailed data (absolute skewness >1 and/or kurtosis >3) to meet normality assumptions. Consequently, follow-up values were set to *NA* if the corresponding baseline values were removed to avoid compromising longitudinal estimates. For this reason, the number of participants included for each analysis slightly differed across cortical features (i.e., listwise deletion). Collinearity was evaluated using the variance inflation factor.

#### Breastfeeding duration, cortical thickness, and surface area

Univariate linear mixed-effects models (LME) were conducted using the *lmerTest* package (version 3.1.3) to assess if variations in breastfeeding duration were associated with differences in cortical thickness or surface area across 148 brain regions, and whether breastfeeding duration moderated the relationship between age (a proxy of time) and these cortical features. Models included fixed effects for household education, handedness, prematurity (preterm yes/no), sex, and a random intercept for participants’ effects. Confounders were selected based on prior association with the brain (see Supplementary Statistical Analysis Section). Total intracranial volume was controlled for in surface area models due to its known influence.^31^ Handedness was removed as a confounder due to its lack of association with the brain. The false discovery rate was controlled separately for breastfeeding’s main effect and breastfeeding-by-age interaction with the Benjamini-Hochberg method.^32^ Statistical significance was considered at corrected *p*-value < 0.05. The model’s syntax can be found below:

$${{{\rm{Cortical}}}}\; {{{\rm{thickness}}}} \sim \,	{{{\rm{breastfeeding}}}}\; {{{\rm{duration}}}} * {{{\rm{age}}}}+{{{\rm{prematurity}}}} \\ 	+{{{\rm{education}}}}+{{{\rm{sex}}}}+(1{{{\rm{|ID}}}})$$

$${{{\rm{Surface}}}}\; {{{\rm{area}}}} \sim \,	{{{\rm{breastfeeding}}}}\; {{{\rm{duration}}}} * {{{\rm{age}}}}+{{{\rm{prematurity}}}}+{{{\rm{education}}}} \\ 	+{{{\rm{sex}}}}+{{{\rm{total}}}}\; {{{\rm{intracranial}}}}\; {{{\rm{volume}}}}+(1{{{\rm{|ID}}}})$$

#### Breastfeeding duration and cortical myelin

Secondary LME models explored the association between breastfeeding duration, breastfeeding-by-age, and cortical myelin in regions where cortical thickness and surface area were associated with breastfeeding duration in the previous analysis. Analyses controlled for prematurity, education, and sex. Statistical significance was considered at uncorrected *p* value < 0.05.

#### Breastfeeding duration and fluid cognition

Additional LME models explored the relationship between breastfeeding duration, breastfeeding-by-age, and fluid cognition in a subsample with cognitive data available and in-person assessments. These analyses controlled for prematurity, education, sex, and vision scores. Statistical significance was considered at uncorrected *p* value < 0.05.

#### Breastfeeding, brain, and fluid cognition (parallel mediation)

This supplementary analysis was conducted at baseline, the visit with the largest number of observations. Prior to testing the parallel mediation, we first confirmed that breastfeeding duration, fluid cognition, and cortical features were all related to multiple regression models (see Supplementary Material for details). Statistical significance was set at uncorrected *p* value < 0.05. To ease interpretability, the cortical features linked to breastfeeding duration and fluid cognition were separately averaged into composites (e.g., cortical thickness composite).

Parallel mediation analyses were conducted using the PROCESS R macro (version 4.3.1). Age, prematurity, education, sex, and vision were considered confounders. Confidence intervals for total, direct, and indirect effects were estimated using 10,000 bootstrap samples. Pairwise contrasts compared the absolute magnitudes of the indirect effects. As the PROCESS macro does not calculate *p-*values for indirect effects, statistical significance was considered if confidence intervals did not include 0.

## Results

The final sample for this study included 5098 individuals at baseline and 3810 at follow-up (see flowchart in the Supplementary Material). At baseline, the average age of participants was 9.92 ± 0.62 years; 48.9% were female (*n* = 2491) and right-handed (80.4%, *n* = 4097). Most participants were White (67.9%, *n* = 3460), non-Hispanic/non-Latino (78.4%, *n* = 3999), and had at least one caregiver with a postgraduate degree (38.2%, *n* = 1949). Regarding breastfeeding duration, most participants were breastfed 1–6 months (*n* = 1766; 34.6%). A detailed description of the sample demographics and other variables of interest at baseline and follow-up can be found in Table 1 and Supplementary Table S1. A comparison of the baseline characteristics of those with available data at follow-up (*n* = 3810) against those with missing data due to COVID-19 restrictions (*n* = 1284) is available in the Supplementary Material section.

**Table 1:** Sample characteristics at baseline and year 2 follow-up visits.

		Baseline (*N* = 5098)		Follow-up, year 2 (*N* = 3810)	
Age in months (mean, sd)		119	7.46	143	7.81
Sex (*n*,%)	Female	2491	48.90%	1831	48.10%
	Male	2607	51.10%	1979	51.90%
Puberty stage (*n*,%)	Pre-pubertal	1320	32.70%	336	10.20%
	Pubertal	2708	67.10%	2898	88.20%
	Post-pubertal	10	0.20%	50	1.50%
Handedness (*n*,%)	Left-handed	343	6.70%	253	6.60%
	Ambidextrous	658	12.90%	494	13%
	Right-handed	4097	80.40%	3063	80.40%
Race (*n*,%)	AI/NA	20	0.40%	16	0.40%
	Asian	74	1.50%	47	1.20%
	Black	637	12.50%	459	12%
	NH/PI	5	0.10%	5	0.10%
	Mixed	609	11.90%	459	12%
	Other race	228	4.50%	159	4.20%
	White	3460	67.90%	2622	68.80%
	Do not know	34	0.70%	20	0.50%
	Refuse answer	14	0.30%	9	0.20%
	Do not answer	18	0.40%	12	0.30%
Ethnicity (*n*,%)	Hispanic/Latinx	1046	20.50%	729	19.10%
	Non-Hispanic/Non-Latino	3999	78.40%	3047	80%
	Do not know	34	0.70%	20	0.50%
	Do not answer	6	0.10%	5	0.10%
	Refuse to answer	13	0.30%	9	0.20%
Mode of delivery (*n*,%)	Vaginal	3253	63.80%	2430	63.80%
	Cesarean section	1844	36.20%	1379	36.20%
	Do not know	1	0%	1	0.10%
Birth issues (*n*,%)	Yes	1193	23.40%	906	23.80%
	No	3776	73.90%	2800	73.50%
	Do not know	139	2.70%	104	2.70%
Pregnancy issues (*n*,%)	Yes	1837	36%	1340	35.20%
	No	3170	62.20%	2405	63.10%
	Do not know	91	1.80%	65	1.70%
Preterm (*n*,%)	On-term	4549	89.20%	3388	88.90%
	Pre-term	449	10.80%	422	11.10%
Preterm group (*n*,%)	Full term (39–40 weeks)	4324	84.80%	3217	84.44
	Early term (37–38 weeks)	225	4.40%	171	4.50%
	Moderate/late preterm (32–36 weeks)	510	10%	395	10.40%
	Very preterm (28–31 weeks)	39	0.80%	27	0.70%
Weeks preterm (mean, sd)	4.59	2.12	4.54	2.06	
Education (*n*,%)	Less than HS	174	3.40%	124	3.30%
	HS/GED	363	7.10%	254	6.70%
	Some college	1221	24%	915	24%
	Bachelor	1391	27.30%	1057	27.70%
	Postgraduate	1949	38.20%	1460	38.30%
Annual income (*n*,%)	< 50,000$	1187	23.3	873	22.90%
	50,000$ to 100,000$	1391	27.30%	1098	28.80%
	>100,000$	2154	42.30%	1584	41.60%
	Refuse to answer	187	3.70%	123	3.20%
	Do not know	179	3.50%	132	3.50%
Breastfeeding duration (*n*,%)	No breastfeeding	805	15.80%	590	15.50%
	1–6 months	1766	34.60%	1.337	35.10%
	7–12 months	1348	26.40%	1026	26.90%
	>12 months	1179	23.10%	857	22.50%

Puberty stage (Pre-pubertal [PDS score of 1], Pubertal [PDS scores between 2 and 4], Post-pubertal [PDS score of 5]). Race (29 items, collapsed into seven groups) and Ethnicity (2 items) were self-reported by the caregiver and only for the interpretation of sample diversity. Birth issues (not requiring hospitalization) refer to any of the following: cyanosis, did not breathe/needed oxygen, slow heartbeat, convulsions, jaundice, required blood transfusion. Pregnancy complications refer to any of the following: severe nausea and vomiting past 2nd trimester or accompanied with weight loss, heavy bleeding requiring bed rest or treatment, pre-eclampsia, eclampsia, toxemia, gall bladder attack, proteinuria, rubella, severe anemia, urinary-tract infections, gestational diabetes, high-blood pressure, previa abruptio, accident or injury requiring medical care.

*AI/NA* American Indian/Native American, *NH/PI* Native Hawaiian/Pacific Islander, *HS* high-school graduate, *GED* generalized education degree.

### Breastfeeding duration, cortical thickness, and surface area

**Fig. 1:**
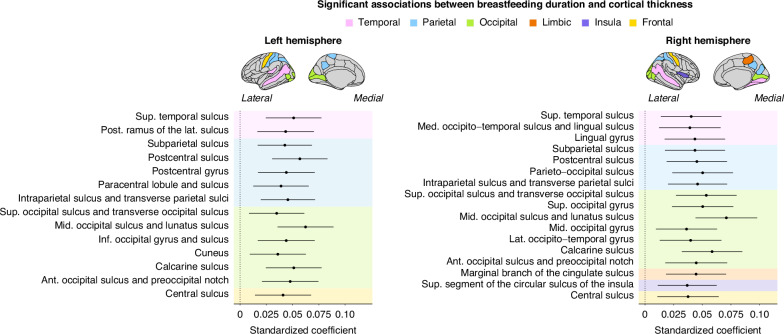
Breastfeeding duration associations with cortical thickness examined using linear mixed-effects models. These models were adjusted for prematurity, education, and sex. Only regions that survived the Benjamini–Hochberg correction are shown. The left panel refers to regions within the left hemisphere. The right panel shows the regions in the right hemisphere. Ant anterior, Post posterior, Sup superior, Inf inferior, Lat lateral, Mid middle, Med Medial, Hor Horizontal.

Significant and positive associations between breastfeeding duration and cortical thickness were found in 31 regions. These posterior regions included lateral (e.g., postcentral and paracentral gyri, superior temporal sulcus) and ventral regions (e.g., cuneus, precuneus, lingual gyrus). The average unstandardized beta coefficient across significant regions indicated that an increase in the breastfeeding duration level (e.g., from no breastfeeding to breastfeeding 1–6 months) corresponded to a 0.006 mm increase in cortical thickness (+0.26%). Regions passing multiple comparison corrections are highlighted in Fig. 1. A table showing the results across all 148 regions is available in Supplementary Table S2. There were no breastfeeding-by-age interactions that passed multiple comparison corrections.

**Fig. 2:**
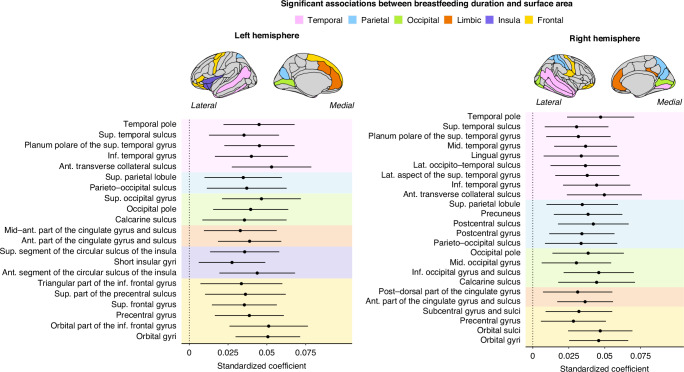
Breastfeeding duration association with surface area examined using linear mixed-effects models. These models were adjusted for prematurity, education, sex, and total intracranial volume. Only regions that survived the Benjamini–Hochberg correction are shown. The left panel refers to regions within the left hemisphere. The right panel shows the regions in the right hemisphere. Ant anterior, Post posterior, Sup superior, Inf inferior, Lat lateral, Mid middle, Med Medial, Hor Horizontal.

There was a significant and positive main effect of breastfeeding duration on the surface area of 45 regions (Fig. 2). Among these, eight regions were located in occipito-parietal regions, overlapping prior findings in cortical thickness. Surface area associations were also observed in anterior regions, such as the prefrontal lobe. For each increase in breastfeeding duration, there was a 9.10 mm^2^ increase in surface area (+0.63%). A detailed description of the results can be found in Supplementary Table S3. No interactions between breastfeeding duration and age passed multiple comparison corrections.

### Breastfeeding duration and cortical myelin

**Fig. 3:**
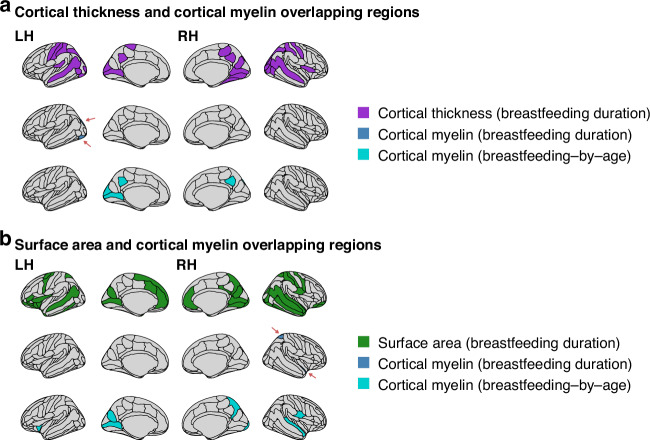
Results from secondary linear mixed-effect models show significant associations between breastfeeding duration and cortical myelin in regions previously showing associations with cortical thickness and surface area. **a** depicts the significant overlapping associations between breastfeeding duration, cortical thickness (purple), and cortical myelin (dark blue). The bottom row of **a** shows the regions where breastfeeding duration moderated the relationship between cortical myelin and age (cyan). **b** shows the significant overlapping associations between breastfeeding duration and surface area (green), cortical myelin (dark blue), and the moderating effect of breastfeeding duration on cortical myelin and age (cyan). RH right hemisphere (row), LH left hemisphere (row).

Among the 31 regions that showed increased cortical thickness with longer breastfeeding duration, the cortical myelin of the left inferior occipital gyrus and sulcus (*β* = 0.032, CI95% = 0.006, 0.059, *p* value = 0. 016) and the left superior occipital sulcus and transverse occipital sulcus (*β* = 0.028, CI95% = 0.002, 0.054, *p* value = 0.038) were positively related to breastfeeding. Breastfeeding duration influenced cortical myelin changes over time in another five posterior regions that did not overlap with the previous result (Fig. 3). That is, those who were breastfed longer exhibited greater cortical myelin by follow-up. The main effects of breastfeeding duration across the 31 regions can be found in Supplementary Table S4. The breastfeeding-by-age interaction results are available in Supplementary Table S5.

Of the 45 regions showing greater surface area relative to longer breastfeeding duration, the cortical myelin of the right superior parietal lobule (*β* = 0. 031, CI95% = 0.005, 0.057, *p* value = 0.018) and the right planum polare of the superior temporal gyrus (*β* = 0.026, CI95% = 0.001, 0.052, *p* value = 0.044) were positively related to breastfeeding duration. Eight regions showed significant positive interactions between breastfeeding duration and age (Fig. 3). Breastfeeding duration effects, as well as its interaction with age, can be found in Supplementary Tables S6 and S7.

### Breastfeeding duration and fluid cognition

The subsample utilized in this analysis included 5006 individuals at baseline and 3006 at follow-up. The characteristics of this subsample were similar to those included in the main analysis and can be found in Supplementary Table S8. While a positive main effect of breastfeeding duration on fluid cognition was found (*β* = 0.06, CI95% = 0.04, 0.09, *p* value < 0.001), no breastfeeding-by-age interaction was observed.

### Breastfeeding duration, brain, and fluid cognition (parallel mediation)

All assumptions to conduct the parallel mediation analysis at baseline were met (see Supplementary Results). Four cortical thickness regions and 23 surface area regions were associated with breastfeeding duration and fluid cognition. No cortical myelin regions showed such associations. Cortical thickness and surface area regions were separately averaged into composites.

**Fig. 4:**
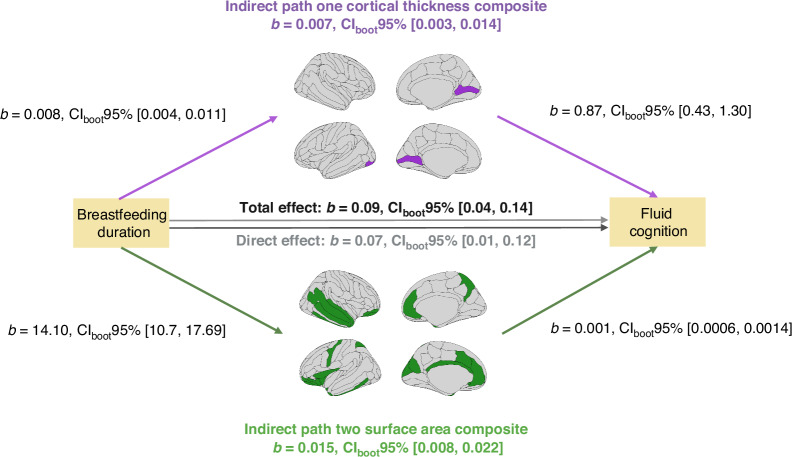
Parallel mediation analysis between breastfeeding duration, fluid cognition, and brain structural correlates. Parallel mediation analysis examining baseline pairwise associations between breastfeeding duration (predictor), fluid cognition (outcome), and cortical thickness composite (mediator 1, 5 regions) and surface area composite (mediator 2, 25 regions), along with the total (breastfeeding duration ◄ fluid cognition), direct (breastfeeding duration ◄ fluid cognition while controlling for mediator 1 and 2), and indirect effects (i.e., breastfeeding duration on fluid cognition via mediator 1 and 2).

The total effect of breastfeeding duration on fluid cognition was statistically significant (*β* = 0.11, CI_boot_95% = 0.051, 0.167, *p* value < 0.001). The direct effect of breastfeeding duration on fluid cognition remained significant after adjusting for cortical thickness and surface area composites (*β* = 0.09, CI_boot_95% = 0.028, 0.145, *p* value = 0.004). The total indirect effect, the sum of cortical thickness and surface area composites, was statistically significant (*β* = 0.023, CI_boot_95% = 0.015, 0.032). Individually, the cortical thickness composite did significantly mediate the relationship between breastfeeding and fluid cognition (*β* = 0.005, CI_boot_95% = 0.002, 0.010), as did the surface area composite (*β* = 0.017, CI_boot_95% = 0.010, 0.025). Pairwise contrasts demonstrated that the indirect effect of the cortical thickness composite was significantly lower than that of the surface area composite (*β *= −0.012, CI_boot_95% = −0.021, −0.003). Figure 4 provides a visual representation of the parallel mediation analysis and results.

## Discussion

This study offers new insights into the association between breastfeeding duration and neurocognitive outcomes during adolescence. Our findings demonstrate that breastfeeding was associated with greater cortical thickness and surface area in a dose-dependent way and that these associations extended into early adolescence. While breastfeeding duration alone had a limited effect on cortical myelin, results showed that cortical myelin changes over time were moderated by breastfeeding duration. Lastly, increased surface area mediated the link between breastfeeding duration and fluid cognition. In sum, our findings emphasize the importance of extending breastfeeding for optimal neurocognitive development.

### Breastfeeding duration, cortical thickness and surface area

Cortical thickness, the distance between white and gray matter surfaces, reflects the number and size of synapses, neurons, and glial cells in the cortex.^33,34^ Our results indicated that longer breastfeeding duration may positively influence cortical thickness in occipital, parietal, and temporal lobes, involved in sensorimotor processing and internally-directed cognition.^33^ While some studies have found similar associations,^9^ others found lower occipital thickness in breastfed boys,^22^ lower parietal thickness in preterm breastfed infants,^19^ or no global changes.^11^ These conflicts may stem from variations in sample size and characteristics, covariate selection, or MRI metrics. Our results suggest lasting benefits of breast milk on cortical growth, which is mainly driven by an overproduction of synapses and glial cells during the first years of life. Neurotrophic factors, choline, and lactoferrin, all present in breast milk, have been related to increased synaptogenesis and gliogenesis and greater neuronal survival.^15,16,35^ Enhanced availability of synapses, glial cells, and neurons may promote resilience and support neurocognitive well-being in later life.^36,37^

Surface area, the extension of the outermost cortical layer, contains local dendrites and axons from deeper layers that facilitate optimal cortico-cortical communications.^33,34^ Our results showed greater surface area in both posterior and anterior regions with prolonged breastfeeding. Research showed that greater surface area in anterior regions is related to enhanced executive function and mental health.^38,39^ Although our findings are partly supported by previous work,^11^ other studies have found no relationships between breastfeeding and surface area.^10,19^ Discrepancies may stem from methodological differences, as one study relied on global averages, potentially obscuring localized effects, and the other focused on preterm infants. Some studies suggest that among preterm individuals, breast milk alone might not provide sufficient nutrition for optimal neurodevelopment, and supplementation with proteins, calcium, and phosphate is advised.^2,40^ Our findings imply that breast milk may enhance early synaptic branching and density across superficial cortical layers, leading to greater surface area, a biomarker for optimal cognition and health.^38,39^

Contrary to our hypothesis, no significant breastfeeding-by-age interactions on cortical thickness and surface area were found. Although cortical thickness and surface area decreased with age following normative trajectories (data not shown), breastfeeding duration did not influence the rate of change in these cortical features. It is possible that the 2-year interval was too narrow or missed the window in which such trajectories diverged. Our results show that breastfeeding’s effects on the brain are cumulative and endure into adolescence, a period of significant brain remodeling,^23^ including synaptic pruning, a key factor in mental health vulnerability.^37^ Optimized cortical integrity may thus help buffer age-related neurocognitive decline, mitigate the effects of psychological distress on neurocognition, and lower the risk of psychiatric disorders developing during adolescence.

### Breastfeeding duration and cortical myelin

While prior research has suggested an association between greater myelination and breastfeeding,^17^ cortical thickness, and surface area,^24,25^ no research has examined to what extent breastfeeding-related changes in these cortical features stem from cortical myelin variations. The left inferior occipital gyrus and sulcus and the right superior parietal lobe showed, respectively, greater cortical thickness and surface area alongside greater cortical myelin. These findings align with evidence that posterior regions are densely myelinated.^33^ Greater myelination, or increased rates of surviving neurons and thereby of axons available for myelination, could be partly due to the impact of breastfeeding and breast milk components. Significant and positive breastfeeding-by-age interactions were found in 12 unique posterior regions previously showing greater cortical thickness (*n* = 5) and surface area (*n* = 8), suggesting that breastfeeding influences on cortical myelin unfold with age. This aligns with evidence that cortical development, including myelination, follows a posterior-to-anterior axis.^33^ This pattern of greater cortical myelin at follow-up may explain the preserved cortical thickness and surface area values observed at this visit.

### Breastfeeding duration and fluid cognition

Our findings also revealed positive associations between breastfeeding duration and fluid cognition, a proxy of executive function critical for academic, professional, and everyday functioning.^26,30^ This association was independent of time, suggesting that the effects of breastfeeding were stable. Although studies relating breastfeeding duration to fluid cognition are scarce, associations between breastfeeding duration and executive function are either limited^21^ or lacking.^20^ It is worth noting that, while correlated, fluid cognition encompasses a broader range of processes than executive function.

### Breastfeeding duration, brain, and fluid cognition (parallel mediation)

Our results at baseline indicated that the positive association between breastfeeding duration and fluid cognition was partly mediated by regions with greater cortical thickness and surface area. In other words, although the original breastfeeding-fluid cognition relationship remained significant, it became weaker after considering the contributions of cortical thickness and surface area. This shows that a proportion of the variance of the breastfeeding-fluid cognition relationship can be explained via the associations between breastfeeding duration, cortical thickness, and surface area. Pairwise comparisons showed that the surface area composite emerged as the strongest mediator. This finding aligns with prior work emphasizing the role of surface area in fluid cognition, as surface area reflects the density of cortico-cortical synapses that facilitate the integration of complex information and, therefore, higher-order cognition.^38^ Moreover, most regions of the surface area composite were located within the prefrontal cortex, a structure that is important for fluid cognition.^41^ It is possible that the improvements in fluid cognition attributed to longer breastfeeding duration are partly due to a greater density of cortico-cortical synapses within the prefrontal cortex.

### Strengths and limitations

The main strength of this study lies in its longitudinal assessment of associations between breastfeeding and neurocognitive outcomes during adolescence in a large, representative sample of youth. This research also presents limitations. It relies on retrospective self-reports of breastfeeding duration. Thus we cannot rule out the possibility of misreporting or recall bias. For example, our study had slightly higher reported rates of breastfeeding in comparison to Diaz et al.^5^ However, this may be explained by the fact that Diaz et al.^5^ did not include participants from Texas, California, Ohio, and other states. California is in the top 10 US states with the highest breastfeeding rates.^42^ In the ABCD Study®, there were four collection sites in California (Stanford Research Institute, UC San Diego, UCLA, and Children’s Hospital Los Angeles). Therefore, regional differences may in part explain the discrepancies in breastfeeding rates between studies. Furthermore, this study lacks information on breastfeeding modality, preventing examination of exclusive breastfeeding vs. mixed feeding (i.e., combining breast and formula-feeding). Additionally, data on breast milk composition were not available, limiting our ability to identify the responsible components for the observed effects. Similar to other research,^43^ the current study could be biased toward participants from high-income families, warranting further replication in samples with different characteristics. Additionally, the 2-year follow-up period may be too short to capture breastfeeding’s full impact on neurodevelopmental trajectories. Also, a moderate number of participants had their year 2 visits postponed because of COVID-19 restrictions,^44^ impacting the availability of neurocognitive data. Additionally, participants from low-income backgrounds may have faced greater challenges during this period,^45^ potentially affecting participation and the generalizability of our findings. Similarly, effects might have occurred outside the developmental windows covered in this study. While T1w/T2w could hint at the density of myelin, its specificity remains debated, as water, intercellular proteins, or iron deposits can influence the T2w signal.^46^ Lastly, while the effect sizes in our study were small, they align with expectations in large-scale studies examining brain development,^47^ especially in relation to distal exposure such as breastfeeding.

## Conclusions

Our findings underscore the potential benefits of breastfeeding on early brain development. Specifically, our results show that longer breastfeeding duration was related to widespread increases in cortical thickness and surface area, key predictors of positive late-life outcomes. Some of these variations correlated with greater cortical myelin, essential for efficient neural communication. Additionally, breastfeeding duration positively influenced fluid cognition, a cognitive capacity predictive of academic, professional, and everyday functioning. This relationship was mediated by breastfeeding-induced changes in surface area, a feature crucial for higher-order cognition. Importantly, these beneficial effects on neurocognition were dose-dependent and stable during a 2-year period covering late childhood and early adolescence, a time known for its intense brain remodeling and increased vulnerability to mental health challenges. Enhanced cortical structure may protect against age-related neurocognitive decline, alleviate psychological distress, and lower the risk of psychiatric disorders emerging during this life stage. Future research should explore specific breast milk components influencing brain structure, examine these relationships across different developmental stages, investigate additional neurobiological mechanisms, and assess how breastfeeding interacts with other influencing factors.

## Supplementary information

Supplementary tables

Supplementary material

## Data Availability

Data used in the preparation of this article were obtained from the ABCD Study® (https://abcdstudy.org/), which is held in the NIMH Data Archive (NDA) and is publicly available. The ABCD Study^®^ data repository grows and changes over time. The ABCD Study^®^ data used in this report were obtained from 10.15154/z563-zd24. The code used to prepare, conduct the analysis, and plot the results can be found in: https://github.com/Adise-lab/abcd-lactation-cth.

## References

[1] Knickmeyer, R. C. et al. A structural MRI study of human brain development from birth to 2 years. *J. Neurosci.* **28**, 12176–12182 (2008). 19020011 10.1523/JNEUROSCI.3479-08.2008 PMC2884385

[2] De Curtis, M. & Rigo, J. The nutrition of preterm infants. *Early Hum. Dev.* **88**, S5–S7 (2012). 22261289 10.1016/j.earlhumdev.2011.12.020

[3] Chiurazzi, M. et al. Human milk and brain development in infants. *Reprod. Med.* **2**, 107–117 (2021).

[4] Horta, B. L., De Sousa, B. A. & De Mola, C. L. Breastfeeding and neurodevelopmental outcomes. *Curr. Opin. Clin. Nutr. Metab. Care* **21**, 174–178 (2018). 29389723 10.1097/MCO.0000000000000453

[5] Diaz, L. E., Yee, L. M. & Feinglass, J. Rates of breastfeeding initiation and duration in the United States: data insights from the 2016–2019 Pregnancy Risk Assessment Monitoring System. *Front. Public Health* **11**, 1256432 (2023). 38192551 10.3389/fpubh.2023.1256432 PMC10773697

[6] Munblit, D., Crawley, H., Hyde, R. & Boyle, R. J. Health and nutrition claims for infant formula are poorly substantiated and potentially harmful. *BMJ* **369**, m875 (2020). 10.1136/bmj.m875 PMC8581741 32376671

[7] Perpiñà Martí, G., Sidera, F., Senar Morera, F. & Serrat Sellabona, E. Executive functions are important for academic achievement, but emotional intelligence too. *Scand. J. Psychol.* **64**, 470–478 (2023). 36843137 10.1111/sjop.12907

[8] Reimann, Z. et al. Executive functions and health behaviors associated with the leading causes of death in the United States: a systematic review. *J. Health Psychol.* **25**, 186–196 (2020). 30230381 10.1177/1359105318800829

[9] Kafouri, S. et al. Breastfeeding and brain structure in adolescence. *Int. J. Epidemiol.* **42**, 150–159 (2013). 10.1093/ije/dys172 23175518

[10] Grevet, L. T. et al. The association between duration of breastfeeding and the trajectory of brain development from childhood to young adulthood: an 8-year longitudinal study. *Eur. Child Adolesc. Psychiatry* **33**, 1863–1873 (2024). 37650992 10.1007/s00787-023-02283-9

[11] Rajagopalan, V., Hsu, E. & Luo S. Breastfeeding duration and brain-body development in 9–10-year-olds: modulating effect of socioeconomic levels. *Pediatr. Res*. **97**, 378–386 (2024). 10.1038/s41390-024-03330-0 PMC11798855 38879625

[12] Lovcevic, I. Associations of breastfeeding duration and cognitive development from childhood to middle adolescence. *Acta Paediatr.* **112**, 1696–1705 (2023). 37166436 10.1111/apa.16837

[13] Keim, S. A. et al. Feeding infants at the breast or feeding expressed human milk: long-term cognitive, executive function, and eating behavior outcomes at age 6 years. *J. Pediatr.* **233**, 66–73.e1 (2021). 33592219 10.1016/j.jpeds.2021.02.025 PMC8154665

[14] Sherzai, D., Moness, R., Sherzai, S. & Sherzai, A. A systematic review of omega-3 fatty acid consumption and cognitive outcomes in neurodevelopment. *Am. J. Lifestyle Med.* **17**, 649–685 (2023). 37711355 10.1177/15598276221116052 PMC10498982

[15] Derbyshire, E. & Obeid, R. Choline, neurological development and brain function: a systematic review focusing on the first 1000 days. *Nutrients* **12**, 1–32 (2020). 10.3390/nu12061731 PMC7352907 32531929

[16] Schirmbeck, G. H., Sizonenko, S. & Sanches, E. F. Neuroprotective role of lactoferrin during early brain development and injury through lifespan. *Nutrients* **14**, 2923 (2022). 35889882 10.3390/nu14142923 PMC9322498

[17] Deoni, S. C., Dean, D., Joelson, S., O’Regan, J. & Schneider, N. Early nutrition influences developmental myelination and cognition in infants and young children. *Neuroimage* **178**, 649–659 (2018). 29277402 10.1016/j.neuroimage.2017.12.056 PMC6540800

[18] Rajhans, P. et al. The role of human milk oligosaccharides in myelination, socio-emotional and language development: observational data from breast-fed infants in the United States of America. *Nutrients* **15**, 4624 (2023). 37960278 10.3390/nu15214624 PMC10649431

[19] Sullivan, G. et al. Breast Milk Exposure is associated with cortical maturation in preterm infants. *Ann. Neurol.* **93**, 591–603 (2023). 36412221 10.1002/ana.26559

[20] Lopez, D. A. et al. Breastfeeding duration is associated with domain-specific improvements in cognitive performance in 9–10-year-old children. *Front. Public Health* **9**, 657422 (2021). 10.3389/fpubh.2021.657422 PMC8109433 33981668

[21] McGowan, C. & Bland, R. The benefits of breastfeeding on child intelligence, behavior, and executive function: a review of recent evidence. https://home.liebertpub.com/bfm; 10.1089/bfm.2022.0192 (2023). 10.1089/bfm.2022.0192 36749962

[22] Li, T. et al. Brain cortical structure and executive function in children may be influenced by parental choices of infant diets. *Am. J. Neuroradiol.* **41**, 1302–1308 (2020). 32527846 10.3174/ajnr.A6601 PMC7357629

[23] Paus, T., Keshavan, M. & Giedd, J. N. Why do many psychiatric disorders emerge during adolescence?. *Nat. Rev. Neurosci.* **9**, 947–957 (2008). 19002191 10.1038/nrn2513 PMC2762785

[24] Natu, V. S. et al. Apparent thinning of human visual cortex during childhood is associated with myelination. *Proc. Natl. Acad. Sci. USA* **116**, 20750–20759 (2019). 31548375 10.1073/pnas.1904931116 PMC6789966

[25] Hill, J. et al. Similar patterns of cortical expansion during human development and evolution. *Proc. Natl. Acad. Sci. USA* **107**, 13135–13140 (2010). 20624964 10.1073/pnas.1001229107 PMC2919958

[26] van Aken, L., Kessels, R. P. C., Wingbermühle, E., van der Veld, W. M. & Egger, J. I. M. Fluid intelligence and executive functioning more alike than different?. *Acta Neuropsychiatr.* **28**, 31–37 (2016). 26281913 10.1017/neu.2015.46

[27] Fischl, B. et al. Automatically parcellating the human cerebral cortex. *Cereb. Cortex* **14**, 11–22 (2004). 14654453 10.1093/cercor/bhg087

[28] Westlye, L. T. et al. Increased sensitivity to effects of normal aging and Alzheimer’s disease on cortical thickness by adjustment for local variability in gray/white contrast: a multi-sample MRI study. *Neuroimage* **47**, 1545–1557 (2009). 19501655 10.1016/j.neuroimage.2009.05.084 PMC2828679

[29] Beer, J. C. et al. Longitudinal ComBat: a method for harmonizing longitudinal multi-scanner imaging data. *Neuroimage* **220**, 117129 (2020). 32640273 10.1016/j.neuroimage.2020.117129 PMC7605103

[30] Akshoomoff, N. et al. NIH toolbox cognition battery (CB): composite scores of crystallized, fluid, and overall cognition. *Monogr. Soc. Res. Child Dev.* **78**, 119–132 (2013). 23952206 10.1111/mono.12038 PMC4103789

[31] Barnes, J. et al. Head size, age and gender adjustment in MRI studies: a necessary nuisance?. *Neuroimage* **53**, 1244–1255 (2010). 20600995 10.1016/j.neuroimage.2010.06.025

[32] Benjamini, Y. & Hochberg, Y. Controlling the false discovery rate: a practical and powerful approach to multiple testing. *J. R. Stat. Soc. Ser. B Methodol.* **57**, 289–300 (1995).

[33] Sydnor, V. J. et al. Neurodevelopment of the association cortices: patterns, mechanisms, and implications for psychopathology. *Neuron* **109**, 2820–2846 (2021). 34270921 10.1016/j.neuron.2021.06.016 PMC8448958

[34] Lyall, A. E. et al. Dynamic development of regional cortical thickness and surface area in early childhood. *Cereb. Cortex* **25**, 2204–2212 (2015). 24591525 10.1093/cercor/bhu027 PMC4506327

[35] Aşkan, Ö. Ö. et al. Unique content of breastmilk: neurotrophic growth factors in breastmilk at 2 years and beyond. *Eur. J. Pediatr.*1–6 https://link.springer.com/article/10.1007/s00431-024-05732-y (2024). 10.1007/s00431-024-05732-y 39207457

[36] Lago-Baldaia, I., Fernandes, V. M. & Ackerman, S. D. More than mortar: glia as architects of nervous system development and disease. *Front. Cell Dev. Biol.* **8**, 611269 (2020). 33381506 10.3389/fcell.2020.611269 PMC7767919

[37] Xie, C. et al. A shared neural basis underlying psychiatric comorbidity. *Nat. Med.* **29**, 1232–1242 (2023). 37095248 10.1038/s41591-023-02317-4 PMC10202801

[38] Tadayon, E., Pascual-Leone, A. & Santarnecchi, E. Differential contribution of cortical thickness, surface area, and gyrification to fluid and crystallized intelligence. *Cereb. Cortex* **30**, 215–225 (2020). 31329833 10.1093/cercor/bhz082 PMC7029693

[39] Fowler, C. H. & Gaffrey, M. S. Reduced cortical surface area globally and in reward-related cortex is associated with elevated depressive symptoms in preschoolers. *J. Affect Disord.* **319**, 286–293 (2022). 36162658 10.1016/j.jad.2022.09.075 PMC13321367

[40] Arslanoglu, S. et al. Fortification of human milk for preterm infants: update and recommendations of the European Milk Bank Association (EMBA) Working Group on human milk fortification. *Front. Pediatr.* **7**10.3389/fped.2019.00076 (2019). 10.3389/fped.2019.00076 PMC6439523 30968003

[41] Østgård, H. F. et al. Executive function relates to surface area of frontal and temporal cortex in very-low-birth-weight late teenagers. *Early Hum. Dev.* **95**, 47–53 (2016). 26939083 10.1016/j.earlhumdev.2016.01.023

[42] Centers for Disease Control and Progression. NIS-Child Breastfeeding Rates by State: 2021. https://www.cdc.gov/breastfeeding-data/about/rates-by-state.html (2024).

[43] Kim, J. Y. et al. The influence of socioeconomic status on individual attitudes and experience with clinical trials. *Commun. Med.* **4**, 172 (2024). 39237734 10.1038/s43856-024-00586-9 PMC11377822

[44] Saragosa-Harris, N. M. et al. A practical guide for researchers and reviewers using the ABCD Study and other large longitudinal datasets. *Dev. Cogn. Neurosci.* **55**, 101115 (2022). 35636343 10.1016/j.dcn.2022.101115 PMC9156875

[45] Yip, S. W., Jordan, A., Kohler, R. J., Holmes, A. & Bzdok, D. Multivariate, transgenerational associations of the COVID-19 pandemic across minoritized and marginalized communities. *JAMA Psychiatry* **79**, 350 (2022). 35138333 10.1001/jamapsychiatry.2021.4331 PMC8829750

[46] Faulkner, M. E. et al. Harnessing myelin water fraction as an imaging biomarker of human cerebral aging, neurodegenerative diseases, and risk factors influencing myelination: a review. *J. Neurochem*. **168**, 2243–2263 (2024). 10.1111/jnc.16170 PMC11951035 38973579

[47] Owens, M. M. et al. Recalibrating expectations about effect size: a multi-method survey of effect sizes in the ABCD study. *PLoS ONE* **16**, e0257535 (2021). 10.1371/journal.pone.0257535 PMC8460025 34555056

