# Supplementary material for: Sustained breastfeeding associations with brain structure and cognition from late childhood to early adolescence
Source: Pediatr Res. 2025 May 17;98(6):2144–52. doi: 10.1038/s41390-025-04086-x (PMC12716639; doi:10.1038/s41390-025-04086-x)
Supplement: Supplementary file 2 — Supplementary material [file 41390_2025_4086_MOESM2_ESM.pdf]

Supplemental Materials

- Supplementary Methods
  - ABCD Study® exclusion criteria — Pg 2
  - Study-specific exclusion criteria — Pg 2
  - Flowchart of participants — Pg 3
  - Demographic and physical characteristics — Pg 3
  - Prenatal/perinatal and pubertal assessments — Pg 4
  - Breastfeeding duration — Pg 5
  - MRI quality control procedures — Pg 6
  - NIH Toolbox® assessments — Pg 6
  - R packages used and confounder selection — Pg 7
  - Parallel mediation analysis assumptions — Pg 7
- Supplemental Results
  - Parallel mediation analysis assumptions — Pg. 8
- Supplementary Tables titles/footnotes (see excel file for the tables) — Pg. 9
- Supplementary discussion — Pg.11
- Supplementary References — Pg 12

Supplementary Methods

1. ABCD Study® exclusion criteria

The ABCD Study had minimal exclusion criteria which included extreme prematurity (born before 28 weeks), neurological or neurocognitive disorders, severe birth complications requiring over 30 days of hospitalization, extremely low birthweight (<1,200 grams), and uncorrectable sensory deficits. Extreme prematurity and extremely low birth weight was cross-referenced with reports at baseline.

2. Study-specific exclusion criteria

To obtain an optimal sample we only included singleton participants (randomly selected) whose biological mother completed the breastfeeding questionnaire. Caregivers that indicated their child had autism spectrum disorder, were intersex, or were prenatally exposed to drugs and alcohol (after knowing of conception), were excluded. Additionally, youth with clinically significant neurological MRI findings (mrif_score = 0, 3 or 4), or had missing or poor-quality MRI data (imgincl_t2w_include = 1) were excluded.

3. Flowchart of participants

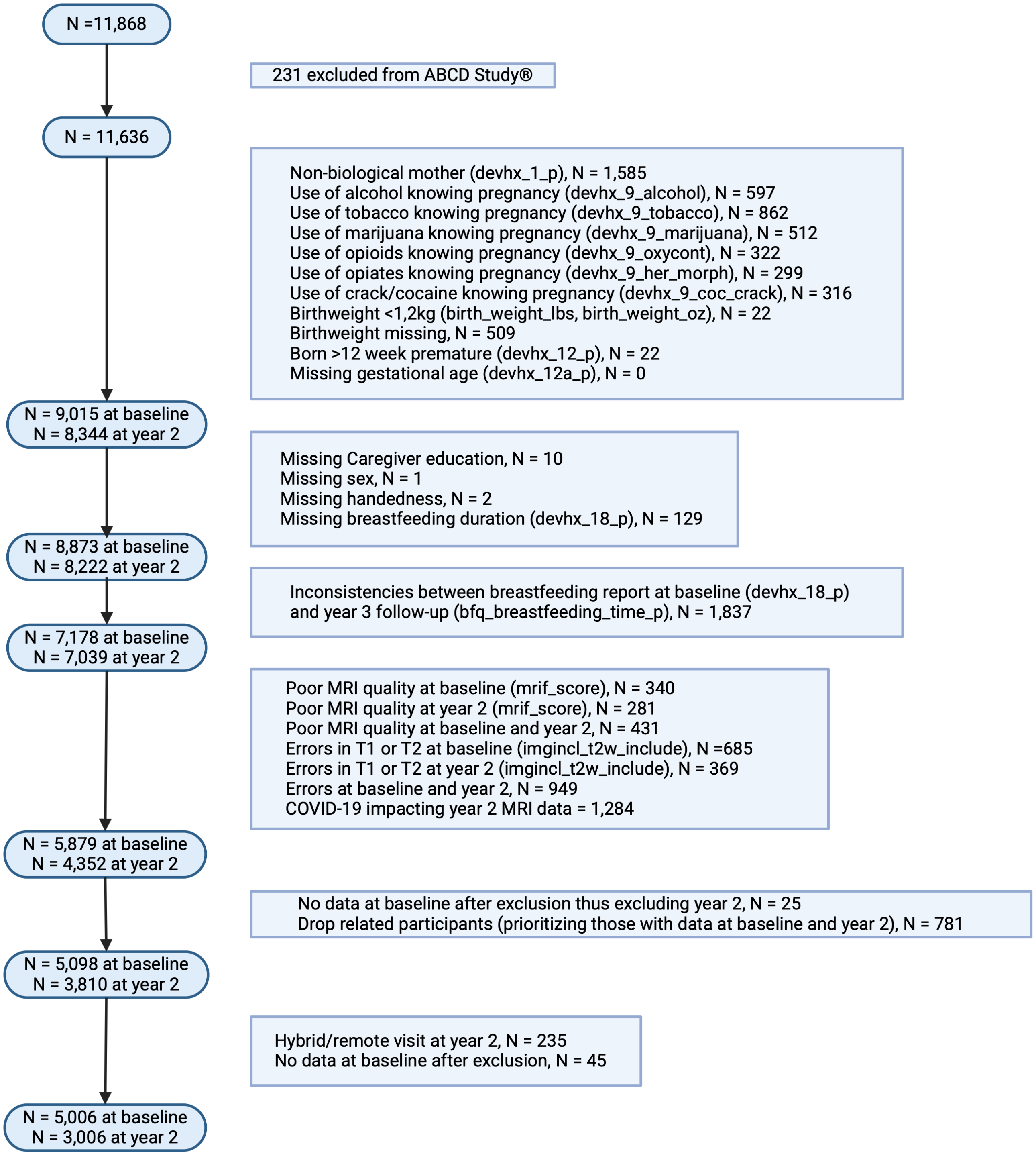

4. Demographic and physical characteristics

Caregivers reported on the youth's race using a 29-item response, which was collapsed into eight categories: American Indian/Native American, Asian, Black, Native Hawaiian/Pacific Islander, Mixed, Other, and White. Caregivers also reported ethnicity as a binary response (Hispanic/Latino, non-Hispanic/Latino). Additional information included the participant's sex assigned at birth (Male/Female), date of birth (used to calculate age at the time of the visit), and household education. Caregiver education was reported on a 21-item scale for all caregivers in the household as follows: less than high school (<13 years), high school graduate (13-14 years), some college (15-17 years), bachelor's degree (18 years), and postgraduate degree (19-21 years). The highest household education level was utilized in the analyses.

At baseline, handedness was assessed using the Youth Edinburgh Handedness Short Form,[1] where participants rated hand preference from -100 (always left) to 100 (always right) for tasks including writing, throwing, brushing their teeth, and using a spoon. Scores above 60 indicated right-handedness, below -60 indicated left-handedness, and scores between -60 and 60 ambidexterity. Visual acuity was assessed using the Snellen Vision Screener,[2] requiring participants to read the smallest line of letters they could see accurately, using corrective lenses if necessary. The last line that was correctly read with both eyes together and no errors determined the vision score.

5. Prenatal/perinatal and pubertal assessments

Prenatal substance exposure (e.g., alcohol, tobacco, marijuana, opiates, opioids, cocaine/crack) before and after pregnancy knowledge was assessed on a binary response (Yes/No) questionnaire at baseline by the attending caregiver. Caregivers also reported on non-exclusionary pregnancy complications (Yes/No), non-hospitalized birth issues (Yes/No), delivery mode (Vaginal/Cesarean), and prematurity (Yes/No).

At each visit, the attending caregiver and the youth completed the Pubertal Development Scale (PDS),[3] which included sex-specific questionnaires assessing growth indicators such as height, body hair, and skin changes. The male version also addressed voice deepening and facial hair, while the female version covered breast development and menstruation, including age of

onset. The PDS consisted of four items with response options scored from 1 (not yet started) to 4 (seems complete), with one item reserved for missingness or *don't know* selections. Menstruation responses were coded as yes or no, with 'yes' corresponding to 4 points. For boys, puberty scores were calculated based on body hair growth, voice deepening, and facial hair, and converted into Tanner stages: pre-pubescent (3), early puberty (>3 and <6), mid puberty (>5 and <9), late puberty (>8 and <12), and post puberty (≥12). For girls, scores were based on body hair growth, breast development, and menarche, with Tanner stages similarly defined. Participants with missing values for any items required for Tanner staging were excluded. To account for potential discrepancies between caregiver and youth reports, an average Tanner stage was calculated from both respondents at each time point. In rare cases where Tanner staging was available from only one responder, the score was derived solely from that report.

6. Breastfeeding duration

At baseline, caregivers retrospectively reported breastfeeding duration in months in an open-ended question at baseline ("*For how many months was your child breast fed?"*). At year 3, caregivers again retrospectively reported breastfeeding duration using an 8-point Likert scale (0 = no breastfeeding, only formula; 1 = several days; 2 = 1-3 months; 3 = 4-6 months; 4 = 7-9 months; 5 = 10-12 months; 6 = 13-18 months; 7 = 19-24 months; 8 = more than 24 months). Given possible discrepancies between baseline and year 3 assessments, breastfeeding reports were cross-referenced to ensure accuracy.

Participants inconsistently reporting no breastfeeding at baseline and year 3 were excluded, (i.e., individuals reporting no breastfeeding at baseline but otherwise at year 3 and vice versa). Additionally, for those reporting some level of breastfeeding at baseline and year 3, we further removed those exhibiting large discrepancies. Large discrepancies were defined as an absolute difference between baseline and year 3 reports of more than 1-unit. In other words, if a caregiver reported at baseline 1-3 months of breastfeeding (response 2) and several days at year 3 (response 1), that participant was kept. If a caregiver reported at baseline 1-3 months of breastfeeding (response 2) and 4-6 months at year 3 (response 3), that participant was kept. However, if a caregiver reported at baseline 1-3 months of breastfeeding (response 2) and 7-9 months at year 3 (response 4), that participant was excluded.

7. MRI quality control procedures

The Data Analysis, Informatics, and Resource Center were responsible for detecting images with artifacts, clinical findings or of poor quality after processing, while the user was responsible for applying appropriate quality control for their study analyses. We excluded data due to FreeSurfer processing failures, T2w post-processing failures, T2w registration to T1w failures, or missing derived results (quality control variable labeled: imgincl_t2w_include). Additionally, we examined the regional values of CT, SA, and CM for unrealistic (e.g., negative values) and out-of-range values (e.g., CT outside its typical range of 1 to 4.5 mm). Participants with values suggesting any of the aforementioned errors were discarded. More information with regard to MRI acquisition and processing can be found elsewhere.[4,5]

8. Fluid cognition

The NIH Toolbox® (http://www.nihtoolbox.org) comprises a series of task-based neurobehavioral assessments designed to evaluate executive function, cognition, emotion, motor skills, and sensory function. In this manuscript, we utilized three specific tasks from the Toolbox:

1. **Flanker Inhibitory Control and Attention Task**: This task assesses executive function by measuring attention skills and the participant's ability to inhibit automatic responses that do not align with their goals (i.e., inhibitory control).
2. **Picture Sequence Memory Task**: This task evaluated visual episodic memory by requiring participants to recall the order of presented images.
3. **Pattern Comparison Processing Speed Task**: This task measured a participant's visual processing speed.

Scores from the NIH Toolbox were reported as uncorrected, age-corrected, or fully corrected t-scores, which are nationally normed across key demographics. In the current manuscript, we focused exclusively on uncorrected task scores for our analyses. To create an alternative composite of fluid cognition, we averaged the scores from these three tasks.

9. Confounder selection

Confounders, including household education,[6] handedness,[7] prematurity,[8] and participant's sex,[9] were selected in accordance with previously established associations with the brain.

10. Parallel mediation assumptions

Prior to testing the parallel mediation, we first confirmed that all statistical assumptions were met. In other words, (1) were breastfeeding duration and fluid cognition associated, (2) were breastfeeding duration and cortical features (i.e., cortical thickness, surface area, cortical myelin) associated, and (3) were the cortical features previously associated with breastfeeding duration also related to fluid cognition. Assumptions were examined with multiple linear regression models at baseline, the visit with the largest number of observations and thus, greater generalizability. As vision acuity can affect cognitive performance, vision scores were considered a confounder. The models' syntaxes are below:

(1) Fluid cognition ~ breastfeeding duration + age + prematurity + education + sex + vision

(2) Cortical features ~ breastfeeding duration + age + prematurity + education + sex (+ total intracranial volume, for SA)

(3) Fluid cognition ~ cortical features + age + prematurity + education + sex + vision

Supplementary Results

1. Parallel mediation analysis assumptions

Linear regression models showed a significant effect of breastfeeding duration on fluid cognition at baseline (b=0.04, CI95%=0.02, 0.07, p-value < 0.001). Among the cortical features associated with breastfeeding duration at baseline, three cortical thickness and 29 surface area regions were also positively associated with fluid cognition at baseline. The cortical myelin of the right frontal superior gyrus was negatively associated with fluid cognition. The three cortical thickness regions and 29 surface area regions were separately averaged into cortical thickness and surface area composites. These composites and the cortical myelin of the right frontal superior gyrus were considered the mediators in the parallel mediation analysis. Supplementary Table S9 provides the results from these analyses.

Supplementary Tables footnotes (see Excel file for the actual tables)

Supplementary Table S1 – Use of drugs before knowing of pregnancy at baseline and year 2 follow-up in the total sample

Footnote: Use of drugs after knowing of pregnancy was "No" for all drugs/substances (exclusion criteria)

Supplementary Table S2 –Linear mixed-effects model results examining the relationship between breastfeeding duration and regional cortical thickness (n = 148)

Footnote: Labels are in accordance with the variable names provided in the 5.1 ABCD data release (note: the N represents the sample size available for the analysis, i.e., outliers were removed to restore normality). L = Left, R = Right, Ant = anterior, Post = posterior, Sup = superior, Inf = interior, Hor = horizontal, Lat = lateral, Med = medial, Mid = middle, Circ = circular, G = gyrus, S = sulcus

Supplementary Table S3 – Linear mixed-effects model results examining the relationship between breastfeeding duration and regional surface area (N = 148).

Footnote: Labels are in accordance with the variable names provided in the 5.1 ABCD data release (note: the N represents the sample size available for the analysis, i.e., outliers were removed to restore normality). L = Left, R = Right, Ant = anterior, Post = posterior, Sup = superior, Inf = interior, Hor = horizontal, Lat = lateral, Med = medial, Mid = middle, Circ = circular, G = gyrus, S = sulcus

Supplementary Table S4 – Linear mixed-effects model results investigating the relationship between breastfeeding duration and regional cortical myelin on cortical thickness regions previously associated with breastfeeding duration (N = 28)

Footnote: Labels are in accordance with the variable names provided in the 5.1 ABCD data release (note: the N represents the sample size available for the analysis, i.e., outliers were removed to restore normality). L = Left, R = Right, Ant = anterior, Post = posterior, Sup = superior, Inf = interior, Hor = horizontal, Lat = lateral, Med = medial, Mid = middle, Circ = circular, G = gyrus, S = sulcus

Supplementary Table S5 – Linear mixed-effects model results investigating the relationship between breastfeeding-by-age and regional cortical myelin on cortical thickness regions previously associated with breastfeeding duration (N = 28)

Footnote: Labels are in accordance with the variable names provided in the 5.1 ABCD data release (note: the N represents the sample size available for the analysis, i.e., outliers were removed to restore normality). L = Left, R = Right, Ant = anterior, Post = posterior, Sup = superior, Inf = interior, Hor = horizontal, Lat = lateral, Med = medial, Mid = middle, Circ = circular, G = gyrus, S = sulcus

Supplementary Table S6 – Linear mixed-effects model results investigating the relationship between breastfeeding duration and regional cortical myelin on surface area regions previously associated with breastfeeding duration (N = 51)

Footnote: Labels are in accordance with the variable names provided in the 5.1 ABCD data release (note: the N represents the sample size available for the analysis, i.e., outliers were removed to restore normality). L = Left, R = Right, Ant = anterior, Post = posterior, Sup = superior, Inf = interior, Hor = horizontal, Lat = lateral, Med = medial, Mid = middle, Circ = circular, G = gyrus, S = sulcus

Supplementary Table S7 – Linear mixed-effects model results investigating the relationship between breastfeeding-by-age and regional cortical myelin on surface area regions previously associated with breastfeeding duration (N = 51)

Footnote: Labels are in accordance with the variable names provided in the 5.1 ABCD data release (note: the N represents the sample size available for the analysis, i.e., outliers were removed to restore normality). L = Left, R = Right, Ant = anterior, Post = posterior, Sup = superior, Inf = interior, Hor = horizontal, Lat = lateral, Med = medial, Mid = middle, Circ = circular, G = gyrus, S = sulcus

Supplementary Table S8 – Baseline and year 2 follow-up characteristics of the subsample with cognitive data available and in-person assessment at year 2

Footnote: Race (29 items, collapsed into 6 groups) and Ethnicity (2 items) were self-reported by the caregiver. AIAN/NHPI = American Indian, Alaska Native/Native Hawaiian, Pacific Islander; HS = High school; GED = Generalized Education Degree; Descriptive statistics are reported for self-reported race only for interpretation of sample diversity. Substance use refers to before knowing of pregnancy (individuals exposed to substances after mother knew of pregnancy were excluded from the current research).

Supplementary Table S9 – Linear regression models examining the relationship between cortical thickness, surface area, cortical myelin with fluid cognition at baseline

Footnote: Labels are in accordance with the variable names provided in the 5.1 ABCD data release (note: the N represents the sample size available for the analysis, i.e., outliers were removed to restore normality). L = Left, R = Right, Ant = anterior, Post = posterior, Sup = superior, Inf = interior, Hor = horizontal, Lat = lateral, Med = medial, Mid = middle, G = gyrus, S = sulcus, CT = cortical thickness, SA = surface area, CM = cortical myelin

Supplementary Table S10 – Comparisons of baseline characteristics of those with data available at follow-up (n = 3,810) against those with missing data due to COVID-19 restrictions (n = 1,284)

Supplementary discussion

Comparisons at baseline suggested that those who were lost at follow-up were approximately 1-month older than those who were lost ($p < 0.001$) and more advanced in puberty ($p < 0.001$). Also, there was a small increase in the number of Asian and Black participants ($p = 0.022$), as well as a higher proportion of Hispanics ($p < 0.001$) that were lost to follow-up. The ABCD Study® had a two-year window for enrollment at baseline, where later enrollment happened to include more youth from historically excluded groups. The results of this comparison can be found in Supplementary Table S10. Due to the timing of enrollment, most of these participants had their two-year visits postponed or missed due to COVID-19 restrictions.[10] Also, there is a chance that these groups may have faced greater challenges during this period,[11] potentially affecting participation and the generalizability of our findings. While slight differences in demographic variables exist, it is important to remark that, except age, none of the variables used in our models, including sex, education, breastfeeding duration, or prematurity, were statistically different between those who continued the study versus those who did not.

Supplementary references

1. Veale JF. Edinburgh Handedness Inventory – Short Form: A revised version based on confirmatory factor analysis. *Laterality: Asymmetries of Body, Brain and Cognition*. 2014;19(2):164-177. doi:10.1080/1357650X.2013.783045
2. Snellen H. *Optotypi Ad Visum Determinandum (Letterproeven Tot Bepaling Der Gezichtsscherpte; Probebuchstaben Zur Bestimmung Der Sehschaerfe)*. Weyers; 1862.
3. Petersen AC, Crockett L, Richards M, Boxer A. A self-report measure of pubertal status: Reliability, validity, and initial norms. *J Youth Adolesc*. 1988;17(2):117-133. doi:10.1007/BF01537962
4. Casey BJ, Cannonier T, Conley MI, et al. The Adolescent Brain Cognitive Development (ABCD) study: Imaging acquisition across 21 sites. *Dev Cogn Neurosci*. 2018;32:43-54. doi:10.1016/j.dcn.2018.03.001
5. Hagler DJ, Hatton SN, Cornejo MD, et al. Image processing and analysis methods for the Adolescent Brain Cognitive Development Study. *Neuroimage*. 2019;202. doi:10.1016/j.neuroimage.2019.116091
6. Norbom LB, Rokicki J, Eilertsen EM, et al. Parental education and income are linked to offspring cortical brain structure and psychopathology at 9–11 years. *JCPP Advances*. 2024;4(1). doi:10.1002/jcv2.12220
7. Sha Z, Pepe A, Schijven D, et al. Handedness and its genetic influences are associated with structural asymmetries of the cerebral cortex in 31,864 individuals. *Proceedings of the National Academy of Sciences*. 2021;118(47). doi:10.1073/pnas.2113095118
8. Kelly CE, Thompson DK, Adamson CL, et al. Cortical growth from infancy to adolescence in preterm and term-born children. *Brain*. 2024;147(4):1526-1538. doi:10.1093/brain/awad348
9. Duerden EG, Chakravarty MM, Lerch JP, Taylor MJ. Sex-Based Differences in Cortical and Subcortical Development in 436 Individuals Aged 4–54 Years. *Cerebral Cortex*. 2020;30(5):2854-2866. doi:10.1093/cercor/bhz279
10. Saragosa-Harris NM, Chaku N, MacSweeney N, et al. A practical guide for researchers and reviewers using the ABCD Study and other large longitudinal datasets. *Dev Cogn Neurosci*. 2022;55:101115. doi:10.1016/j.dcn.2022.101115
11. Yip SW, Jordan A, Kohler RJ, Holmes A, Bzdok D. Multivariate, Transgenerational Associations of the COVID-19 Pandemic Across Minoritized and Marginalized Communities. *JAMA Psychiatry*. 2022;79(4):350. doi:10.1001/jamapsychiatry.2021.4331
